# APPLY: A prospective observational study of clinical practice patterns of darbepoetin alfa use in patients with chemotherapy-induced anemia in Romania

**DOI:** 10.1007/s12254-018-0390-4

**Published:** 2018-03-02

**Authors:** Nicoleta Mariana Berbec, Dana Lucia Stanculeanu, Nicoleta Sorina Badelita, Mariana Vasilica, Dorel Ionel Popovici, Andrei Colita, Cristina Neacsu, Alexandru Iordan

**Affiliations:** 1 Colţea Clinical Hospital, 1 Ion C. Brătianu Bd, Bucharest, Romania; 2 Institute of Oncology “Prof. Dr. Al Trestioreanu”, 252 Fundeni Rd, 2nd District, Bucharest, Romania; 3 Fundeni Clinical Institute, 258 Fundeni Rd., Bucharest, Romania; 4 OncoHelp Clinic, 59 Ciprian Porumbescu St., Timisoara, Romania; 5 Amgen Romania, 1A Bucuresti-Ploiesti Rd., 1st District, Bucharest, Romania

**Keywords:** Darbepoetin alfa, Chemotherapy-induced anemia, Hemoglobin, Fatigue, EORTC guidelines, Romanian National Therapeutic Protocol on ESA prescription

## Abstract

**Purpose:**

The primary objective of this study was to evaluate the compliance of Romanian physicians with the national therapeutic protocol and international guidelines on treatment with erythropoiesis-stimulating agents in anemic cancer patients receiving chemotherapy. The secondary objective was to assess the hemoglobin (Hb) level change due to anemia treatment and safety of darbepoetin alfa.

**Methods:**

This was a single-arm, prospective, longitudinal, multicenter, observational study in patients with nonmyeloid malignancies and symptomatic chemotherapy-induced anemia treated concomitantly with darbepoetin alfa. Patients were followed for the duration of chemotherapy, but no shorter than three and no longer than eight cycles, irrespective of their exposure to darbepoetin alfa.

**Results:**

In this study, 497 patients with a mean age of 60.6 years were analyzed. Most patients (80.7%) were initiated on darbepoetin alfa at a Hb of 9–11 g/dL, congruent with recommendations. The median Hb increased by 0.9 g/dL between baseline and week 12. Hb target achievement was higher among patients treated according to guidelines than those initiated at Hb < 9 g/dL. A similar trend was observed for red blood cell transfusion requirements. No new safety signals were reported for darbepoetin alfa.

**Conclusions:**

The majority of patients were treated according to national and international recommendations. Guideline adherence was associated with more frequent achievement of Hb targets and lower red blood cell transfusion requirements compared with patients starting anemia treatment with darbepoetin alfa at lower-than-recommended Hb levels.

**Electronic supplementary material:**

The online version of this article (10.1007/s12254-018-0390-4) contains supplementary material, which is available to authorized users.

## Introduction

Chemotherapy-induced anemia, a common complication of myelotoxic chemotherapy [1, 2], is associated with clinical symptoms such as fatigue, which seriously compromises patients’ quality of life [3]. Guidelines commonly recommend the administration of erythropoiesis-stimulating agents (ESAs) to improve patients’ quality of life and to avoid severe, life-threatening anemia, which can only be alleviated with the help of allogeneic red blood cell transfusions (RBCTs) [4, 5]. Darbepoetin alfa, a long-acting ESA, is authorized to be administered subcutaneously to patients with symptomatic chemotherapy-induced anemia (hemoglobin [Hb] concentration ≤10 g/dL) at a recommended initial dose of 500 μg (6.75 μg/kg) given once every three weeks. Once weekly dosing can be given at 2.25 μg/kg body weight. If the clinical response of the patient (fatigue, Hb response) is inadequate after 9 weeks, further therapy may not be effective. In the European Medicines Agency (EMA) Summary of Product Characteristics (SmPC), Hb concentrations should not exceed 12 g/dL, although due to intrapatient variability, occasional individual Hb values for a patient above and below the desired level may be observed and the darbepoetin alfa dose should be managed so that a Hb target range of 10–12 g/dL is maintained [6].

In accordance with international guidelines, the goal of anemia treatment as per the Romanian National Therapeutic Protocol on ESA prescription is to reduce the requirement for allogeneic RBCTs and maintain quality of life in patients receiving chemotherapy [7]. According to the Romanian National Therapeutic Protocol on ESA prescription at the time of study design, a Hb concentration of 9–11 g/dL was recommended for the initiation of ESA therapy. In 2013, the local prescription protocol in Bucharest changed and allowed initiation of ESAs only in patients with a Hb in the range of 8–10 g/dL, drawing on European Society of Medical Oncology (ESMO) recommendations with a cut-off Hb of ≤10 g/dL for ESA initiation [5]. However, there are no data available on how well these instructions are followed and implemented in routine clinical practice in Romania. Therefore, the aim of this long-term prospective study was to assess how treatment standards for chemotherapy-induced anemia have evolved over time in Romania, to describe how effectively patients with chemotherapy-induced anemia are treated with darbepoetin alfa, and to examine adherence to authority recommendations and product labeling.

## Methods

### Study design

This was a single-arm, longitudinal, multicenter observational (noninterventional) study, prospectively collecting data from patients with nonmyeloid malignancies suffering from symptomatic chemotherapy-induced anemia, who were treated with darbepoetin alfa. Patients were treated and observed over a favorable minimum of 3 and a maximum of 8 chemotherapy cycles.

Patient data were taken from the medical files and registered in an electronic web-based documentation form. Data were collected at enrollment and during chemotherapy treatment for up to 4 weeks after the latest dose of darbepoetin alfa. In case chemotherapy was discontinued, darbepoetin alfa was discontinued approximately 4 weeks after the end of chemotherapy (as per SmPC) and patient data were collected up to 4 weeks after the last dose of darbepoetin alfa.

This noninterventional study did not alter the routine clinical management of the participating patients and no laboratory or diagnostic tests, other than those performed as part of the patient’s routine medical care, were required.

### Study objectives and endpoints

The primary study objective was to evaluate the compliance with the local Romanian national therapeutic protocol and with European Organization for Research and Treatment of Cancer (EORTC) guidelines on ESA treatment. The primary endpoint was to estimate the proportion of patients within the Hb range of 9–11 g/dL at initiation of darbepoetin alfa.

Secondary objectives included the assessment of Hb level change over time and the safety of darbepoetin alfa. Secondary endpoints were the proportion of darbepoetin alfa-treated patients achieving a Hb concentration >11.0 g/dL during the study at weeks 6, 9, and 12; the median increase in Hb from initiation of darbepoetin alfa to week 6, 9, 12 and the last observed Hb level; the proportion of darbepoetin alfa treated patients (with a Hb < 10.0 g/dL at darbepoetin alfa initiation) achieving a Hb concentration ≥10.0 g/dL during the study at weeks 6, 9, and 12; the time to and the mean Hb concentration after achieving first time Hb level ≥10.0 g/dL after darbepoetin alfa initiation; the proportion of achieved Hb levels at weeks 6, 9, 12, and the last observed Hb level compared to baseline Hb levels; the proportion of patients treated with darbepoetin alfa with at least one RBCT or full blood transfusion during the study; the RBCT or full blood transfusion requirements per baseline Hb initiated during the treatment period with darbepoetin alfa 500 μg once every 3 weeks (Q3W; estimation of the number of transfusions per patient, transfusion rates by both crude rate and Kaplan–Meier method, as well as number of transfusion units administered during the treatment period); cumulative dose and duration of dosing of darbepoetin alfa; and a summary of darbepoetin alfa treated patients receiving computed tomography (CT) at Full Dose on Schedule (FDOS). A cycle was considered to be full dose on schedule if percent of dose reduction (DR) of any myelotoxic drug did not exceed 10% of planned amount and dose delay from planned date (if any) did not exceed 3 days. Adverse drug reactions (ADRs) to darbepoetin alfa were also collected.

### Eligibility criteria

Patients with a diagnosis of solid or hematological tumors (nonmyeloid malignancies), regardless of their disease status, who received chemotherapy and experienced chemotherapy-induced anemia with one or more symptoms of fatigue and who received at least one dose of darbepoetin alfa before enrollment were included. Darbepoetin alfa needed to be administered in accordance with the version of the SmPC valid at the time of study conduct. Patients had to have a life expectancy of at least 4 months and had to sign an informed consent form.

Patients were excluded if they had any contraindication for treatment with darbepoetin alfa as per SmPC or if they concurrently participated in any other clinical trial, were administered any ESA therapy other than darbepoetin alfa or had received RBCTs or full-blood transfusion up to 28 days prior to enrollment, or had planned radiotherapy during treatment with darbepoetin alfa.

### Statistical analysis

A formal hypothesis was not tested. Statistical analyses were descriptive in nature. For continuous variables, the mean, standard deviation (SD), median, first and third quartiles (interquartile range, IQR), minimum and maximum values were presented along with 95% two-sided confidence intervals (CIs), where appropriate. Missing values of continuous variables were counted as “missing”. However, to assess Hb level at week 6, week 9, and week 12 last observation carried forward (LOCF) rule or closest value imputation rule was applied. For categorical variables, the number and percentage of patients in each category were reported. Missing values for categorical variables were excluded from the calculation of CIs, but the number and percentage of patients with missing results were provided. For binary variables, the number and percentage of patients were reported, along with exact two-sided CIs, where appropriate.

The analysis of all predefined variables, including safety, was performed on the full analysis set (FAS). The FAS consisted of all patients who met the eligibility criteria and received at least one dose of darbepoetin alfa. Analyses were repeated for the per-protocol analysis set, defined as all patients who met the eligibility criteria, received darbepoetin alfa for symptomatic chemotherapy-induced anemia, and completed a minimum of 3 and up to a maximum of 8 cycles of chemotherapy, irrespective of the length of their exposure to darbepoetin alfa.

To determine the sample size necessary to assess the proportion of patients with Hb concentrations within the range 9–11 g/dL at initiation of darbepoetin alfa, previous observation studies were taken as a basis. Previous studies showed a proportion of patients with Hb concentrations in the range of 9–11 g/dL at enrollment of about 64%. A sample size of 500 patients was found to ensure that the half-width of the 95% CI for the proportion of patients with Hb at enrollment in the range of 9–11 g/dL would be no larger than 4.4 percentage points (p. p.). The calculated sample size also allowed getting results with sufficient precision in all other endpoints.

Following the change in the Romanian protocol to use ESAs according to current label, a supplemental statistical analysis plan was designed to further assess the adherence to the current darbepoetin alfa label as well as to assess the efficacy results if darbepoetin alfa was used in patients with Hb levels of 8–10 g/dL. Post hoc the data were also assessed separately for patients with solid tumors and hematological malignancies.

## Results

**Fig. 1:**
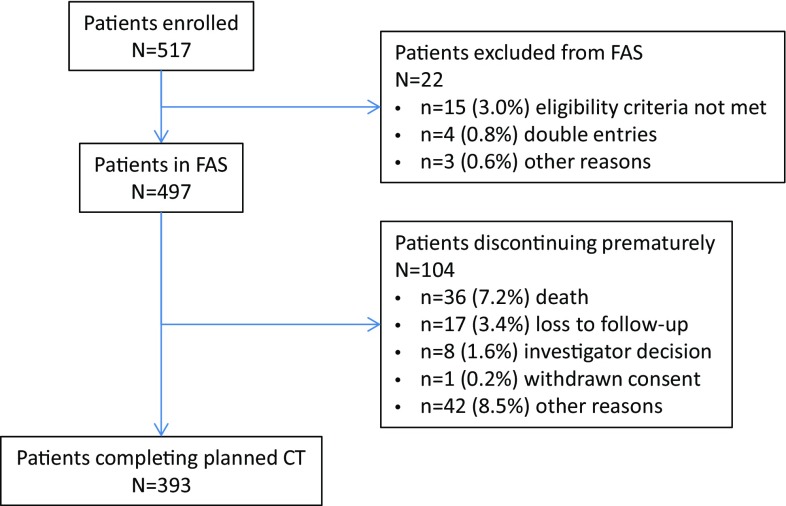
Patient disposition. Percentages are based on the number of patients in the FAS. *CT* chemotherapy, *FAS* full analysis set

The study was conducted between December 2011 and February 2015 in 35 Romanian centers, with a total study duration of 38 months. The FAS comprised 497 eligible patients; 22 patients were excluded from statistical analysis. Of the patients included in the FAS, 393 (79.1%) completed 3–8 chemotherapy cycles as planned; 104 patients (20.9%) discontinued (Fig. 1). The median (IQR) duration of observation was 4.0 (3.0, 5.0) cycles and 107.0 (80.0, 140.0) days.

Patient mean (SD) age was 60.6 (10.67) years and all patients were of Caucasian ethnicity. The vast majority of patients (*n* = 483; 97.2%) had an Eastern Cooperative Oncology Group (ECOG) performance status of 0–2. Patients had a variety of solid or hematological cancers. The median (IQR) time since diagnosis was 107.0 (62.0, 278.0) days. The majority of patients had no comorbidities prior to enrollment and had received no prior anticancer therapies or radiotherapy (Table 1). Most patients had not received any anemia treatments prior to study enrollment: 479 patients (96.4%) had not received prior iron therapy and 473 (95.2%) had not received any RBCT or full blood transfusions. Baseline laboratory assessments relevant to anemia are shown in Table S1 in the online supplemental material.

**Table 1:** Patient demographics and baseline characteristics

Characteristics	*N* = 497
*Age*	
Mean (SD), years	60.6 (10.67)
Median (Q1, Q3), years	62.0 (55.0, 68.0)
Min–Max, years	23–84
<65 years, *n* (%)	319 (64.2)
≥65 years, *n* (%)	178 (35.8)
*Gender, n (%)*	
Male	255 (51.3)
Female	242 (48.7)
*Performance status (ECOG), n (%)*	
0	51 (10.3)
1	278 (55.9)
2	154 (31.0)
3	11 (2.2)
N/A	3 (0.6)
*Tumor type, n (%)*	
Lung cancer	98 (19.7)
Breast cancer	39 (7.8)
Gynecological tumors	57 (11.5)
Other solid tumors	145 (29.2)
Hodgkin’s disease	6 (1.2)
Multiple myeloma	56 (11.3)
Non-Hodgkin’s lymphoma	78 (15.7)
Other hematological malignancies	18 (3.6)
*Time since diagnosis*	
*N*	453
Mean (SD), days	314.4 (597.38)
Median (Q1, Q3), days	107.0 (62.0, 278.0)
Min–Max, days	3–6816
Missing, *n*	44
*Any comorbidities prior to enrollment, n (%)*	108 (21.7)
*Any anticancer therapy prior to enrollment, n (%)*	44 (8.9)
*Radiotherapy prior to enrollment, n (%)*	36 (7.2)

*ECOG* Eastern Cooperative Oncology Group, *max* maximum value, *min* minimum value, *n* number of patients with the given parameter, *N* total number of patients in the group/subgroup, *SD* standard deviation, *Q1* lower quartile, *Q3* upper quartile

### Adherence to guidelines and hemoglobin evolution over time

At initiation of darbepoetin alfa, 401 patients (80.7%, 95% CI 76.9, 84.1) had a Hb within the range of Hb 9–11 g/dL recommended by the EORTC and the Romanian guidelines (primary endpoint; Table 2). These findings were independent of age category (<65 or ≥65 years), gender, or chemotherapy type, but differed by certain tumor types (Table S2). The approved label recommends a Hb ≤ 10 g/dL at darbepoetin alfa initiation, which was adhered to by 269 patients (54.1%, 95% CI 49.6, 58.6). For differences by age, gender, chemotherapy type, tumor type and FDOS, see Table S3.

**Table 2:** Hb categories at baseline and RBCT requirements by Hb category and tumor type

	Patients in category	Required transfusion		Time to first transfusion
	*n* (%)	*n*/*N* (%)	95% CI	Median (IQR), days
*Overall*	497 (100.0)	67 (13.5)	–	–
Hb < 9 g/dL	77 (15.5)	16 (20.8)	12.4, 31.5	38.5 (21.5, 71.5)
Hb 9–11 g/dL	401 (80.7)	50 (12.5)	9.4, 16.1	64.0 (34.0, 93.0)
Hb > 11 g/dL	19 (3.8)	1 (5.3)	0.1, 26.0	131.0 (131.0, 131.0)
*After 5 weeks of darbepoetin alfa treatment*				
Overall	497 (100.0)	47/472 (10.0)	7.4, 13.0	–
Solid tumors	339 (100.0)	29/320 (9.1)	6.2, 12.8	–
Hematological malignancies	158 (100.0)	19/152 (11.8)	7.2, 18.1	–

For RBCT requirements by Hb category the entire study period was taken into consideration and the proportions are based on patients in the FAS. For RBCT requirements by tumor type, the period after 5 weeks of treatment with darbepoetin alfa was taken into consideration and proportions are based on the number of patients still on study after week 5

*CI* confidence interval, *n* number of patients with the given parameter, *N* total number of patients in the group/subgroup, *RBCT* red blood cell transfusion, *Hb* hemoglobin, *IQR* interquartile range

**Fig. 2:**
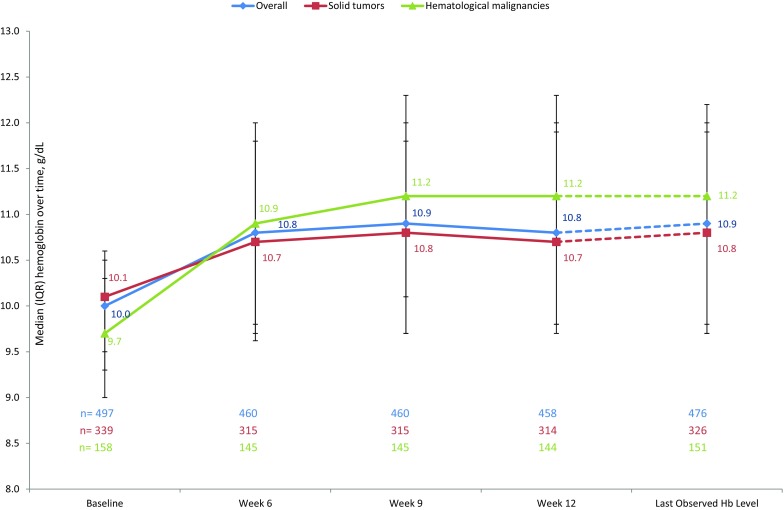
Median (IQR) hemoglobin over time, overall and by tumor type. The timepoint of last observed Hb is different for each patient; the dotted line shows that last observed Hb was not necessarily measured after week 12. *IQR* interquartile range, *Hb* hemoglobin

Hb target achievement rates were provided for two different Hb levels as Hb ≥ 10 and Hb > 11 g/dL. The proportions of patients with Hb < 10 g/dL at baseline achieving Hb ≥ 10 g/dL at weeks 6, 9, and 12 were 58.6% (*n* = 133/227), 60.0% (*n* = 137/226), and 56.5% (*n* = 126/223), respectively (Table 3). After achieving ≥10 g/dL for the first time, patients had a median (IQR) Hb of 10.96 (10.19, 11.93) g/dL. The proportions of patients with Hb ≤ 11 g/dL at baseline achieving Hb > 11 g/dL at weeks 6, 9, and 12 were 42.4% (*n* = 188/443), 46.0% (*n* = 204/443), and 44.7% (*n* = 197/441; Table 3). After achieving Hb > 11 g/dL for the first time, patients had a median (IQR) Hb of 11.70 (10.90, 12.30) g/dL. The median (IQR) time to achieve Hb ≥ 10 g/dL was 30.0 (21.0, 47.0) days; the median (IQR) time to achieve Hb > 11 g/dL was 34.5 (21.0, 57.0) days (Table S4). More patients with hematological malignancies than with solid tumors achieved Hb targets (Table 3). There was no significant difference by age category or gender. More patients receiving anthracyclines or regimens based on new monoclonal antibodies achieved these Hb target categories than other chemotherapy types (trend estimated for groups with a minimum of 20 patients at week 6). Fig. 2 shows Hb trajectories, overall and by tumor type. The shift categories from baseline to week 12 in Table 4 show the proportion of patients achieving a certain Hb value based on Hb at initiation. Of patients who were initiated on darbepoetin alfa at Hb < 9 g/dL, 26.8% achieved Hb > 11 g/dL; in patients initiated at Hb 9–11 g/dL, 48.1% achieved Hb > 11 g/dL.

**Table 3:** Proportion of patients achieving Hb ≥ 10 or >11 g/dL at specific timepoints

	Overall			Solid tumors			Hematological malignancies		
Covariates	*N*	*n* (%)	95% CI	*N*	*n* (%)	95% CI	*N*	*n* (%)	95% CI
*Patients achieving Hb* *≥* *10* *g/dL*									
Week 6	227	133 (58.6)	51.9–65.1	133	71 (53.4)	44.5–62.1	94	62 (66.0)	55.5–75.4
Week 9	226	137 (60.6)	53.9–67.0	133	72 (54.1)	45.3–62.8	93	65 (69.9)	59.5–79.0
Week 12	223	126 (56.5)	49.7–63.1	131	68 (51.9)	43.0–60.7	92	58 (63.0)	52.3–72.9
*Patients achieving Hb* *>* *11* *g/dL*									
Week 6	443	188 (42.4)	37.8–47.2	315	130 (41.3)	35.8–46.9	137	65 (47.4)	38.9–56.1
Week 9	443	204 (46.0)	41.3–50.8	315	135 (42.9)	37.3–48.5	137	73 (53.3)	44.6–59.4
Week 12	441	197 (44.7)	40.0–49.4	314	135 (43.0)	37.4–48.7	137	69 (50.7)	42.0–59.4

Percentages are based on the number of patients in the specified subgroup in Full Analysis Set who have Hb concentration <10 and ≤11 g/dL, respectively, at baseline and at least one nonmissing value at a given timepoint. Hb values within 28 days after RBC or full blood transfusion were excluded from the analysis. Confidence intervals are obtained from the exact method

*CI* confidence interval, *Hb* hemoglobin, *n* number of patients with the given parameter, *N* total number of patients in the group/subgroup

**Table 4:** Shift from baseline in Hb levels at week 12 (proportions of patients)

Hb level at week 12	Hb level at Baseline (in %)			
	<9 g/dL	9–11 g/dL	>11 g/dL	Total
<10 g/dL	50.7	28.9	5.9	31.4
≥10 g/dL	49.3	71.1	94.1	68.6
≤11 g/dL	73.2	51.9	29.4	54.4
>11 g/dL	26.8	48.1	70.6	45.6

Proportions are based on the total *N* in each baseline Hb category

*Hb* hemoglobin, *N* total number of patients in the group/subgroup

### Transfusion requirements

Overall, 13.5% of patients (*n* = 67) had a total of 221 transfusions, 178 (80.5%) of which were packed RBCTs and 43 (19.5%) were full blood transfusions. Of 472 patients remaining on study after 5 weeks, 47 (10.0%) had received their transfusion after a period of 5 weeks of darbepoetin alfa treatment. Of patients with a baseline Hb < 9 g/dL, 20.8% (*n* = 16/77) required transfusions, as did 12.5% (*n* = 50/401) with a baseline Hb of 9–11 g/dL, and 5.3% (*n* = 1/19) with a baseline Hb > 11 g/dL. The median time to first transfusion was 38.5 days in the baseline Hb category <9 g/dL, 64.0 days in the Hb category 9–11 g/dL, and 131.0 in the Hb category >11 g/dL (Table 2). Per transfusion event, ≤2 units were administered in 218 (98.6%) events and >2 units in 3 (1.4%) events.

### Chemotherapy delivery

According to the described definition, 30.0% of patients (*n* = 149, 95% CI 26.0, 34.2) received FDOS. There was, however, some difference between chemotherapy and tumor types as detailed in Table S5.

### Exposure to darbepoetin alfa

The median (IQR) cumulative administered darbepoetin alfa dose was 1800.0 (1000.0, 2500.0) µg (range 500–8000), with a median (IQR) duration of exposure of 84.0 (53.0, 117.0) days (range 7–352).

### Safety

One non-serious ADR (vena cava thrombosis) and no serious ADRs were reported.

## Discussion

Recommendations for ESA initiation vary between international guidelines, with the EORTC guidelines recommending a Hb range of 9–11 g/dL [4] and the ESMO guidelines recommending Hb ≤ 10 g/dL [5]. Romanian health authorities recommended the initiation of ESA therapy in patients with a Hb range of 9–11 g/dL and later on in patients with Hb level between 8–10 g/dL [7]. The APPLY study (A Prospective Aranesp® Observational Study in Patients with Chemotherapy-Induced Anemia) found that 80.7% of patients were initiated on darbepoetin alfa treatment within the Hb range of 9–11 g/dL, indicating that treatment of patients with chemotherapy-induced anemia is generally congruent with the Romanian recommendations. The darbepoetin alfa label recommendations of Hb ≤ 10 g/dL at initiation were adhered to by 54.1%.

There is only a limited number of studies reporting Hb strata at initiation of ESAs generally, and of darbepoetin alfa in particular, and reported strata vary between studies because of the above-mentioned differences in recommendations and a label change in 2008, where the Hb level recommended for initiation of ESAs as changed from Hb 9–11 g/dL to ≤10 g/dL. Of noninterventional studies, the international IMPACT study (A Retrospective and Prospective Observational Study Reviewing Supportive Care Management of NHL Patients Treated With CHOP-14 or 21 Chemotherapy With or Without Rituximab) was conducted in 14 European countries and Australia [8]. In the subanalysis of patients with non-Hodgkin lymphoma receiving cyclophosphamide–doxorubicin–vincristine–prednisolone (CHOP) ± rituximab, 67% of patients had Hb concentrations of 9–11 g/dL at the time of initiation of darbepoetin alfa [8]. The observational, noninterventional ‘A Prospective Data Audit from Patients Treated with Aranesp Due to Chemotherapy Induced Anaemia’ (APRIORI) study conducted in 6408 patients in Poland, The Czech Republic, Slovakia, Slovenia, Hungary, and Russia assessed adherence to treatment guidelines for the use of darbepoetin in patients with chemotherapy-induced anemia, i. e., adherence to product labeling recommendations before and after changes were made to the darbepoetin alfa SmPC in 2008. The study found that 56.4% of patients were initiated on darbepoetin alfa at a Hb in the range of 9–11 g/dL range dL before label change versus 90.0% of patients initiating darbepoetin alfa at a Hb ≤ 10 g/dL after label change [9]. Compared to these studies, in APPLY substantially more patients had darbepoetin alfa initiated in the Hb range of 9–11 g/dL, which is in line with Romanian health authority guidance and EORTC guidelines.

In APPLY, which to our knowledge is the largest observational with darbepoetin alfa in Romania, symptomatic anemia was successfully corrected with a median Hb increase of 0.90 g/dL from a median Hb of 10.00 g/dL at baseline to 10.80 g/dL at week 12. Change from baseline was higher in patients with hematological malignancies than in those with solid tumors. The APPLY study descriptively analyzed Hb target achievement in two ways: achievement of Hb > 10 g/dL and achievement of Hb > 11 g/dL. Interestingly, if anemia treatment was initiated at a baseline Hb within the recommended range of 9–11 g/dL substantially more patients achieved either of these targets compared to patients who were initiated with darbepoetin alfa at a Hb value <9 g/dL. The median time to achieve Hb > 10 g/dL or Hb > 11 g/dL was 30.0 days and 34.5 days, respectively. Of note, Hb values within 28 days after RBCT or full blood transfusion were excluded from the analysis; therefore, transfusions did not alter the results. This is in line with the IMPACT study, where 52% of patients with a baseline Hb < 10 g/dL achieved a Hb ≥ 10 g/dL after 5 weeks of treatment with darbepoetin alfa, and 44% of patients with Hb < 11 g/dL achieved Hb ≥ 11 g/dL after the same period [8]. In the European observational study ‘Current Practice of Aranesp in the Management of Hemoglobin Levels: an Observational International Cancer Evaluation’ (CHOICE), conducted in 11 European countries in 1900 patients with solid tumors, 57% of enrolled patients had a baseline Hb level <10 g/dL and 91% had <11 g/dL. At week 9, 54% of patients still in the study and with available Hb values achieved the predefined Hb target value of 10–12 g/dL [10, 11]. Taken together the findings of the present study are in line with other observational studies conducted in Europe.

Transfusion requirements differed by Hb category at baseline. Patients who were initiated with darbepoetin alfa at a Hb within the recommended range of 9–11 g/dL requiring fewer transfusions compared with patients initiated below the recommended range. Patients treated according to the recommendations also had a longer time to first transfusions than patients initiated at a lower than recommended Hb. A study estimating the incidence of at least one transfusion by baseline Hb ranges found that transfusion rates were highest in the <9 g/dL baseline Hb group in all 4‑week time periods examined [12]. Deger et al. showed similar trends, reporting an association between Hb level at initiation of darbepoetin alfa, transfusion requirements and associated costs for transfusion [13].

This study has several limitations. The primary risk of bias in observational studies is that of selection bias, wherein the sampled patient population may not be representative of the overall patient population in the region. Also, only patients treated with darbepoetin alfa were included. The decision to enroll a given patient into the study was taken after the physician had decided on the individual treatment approach for a given patient. It is unclear whether this may have introduced a certain selection bias, since longer-acting ESAs may be selected for different types of patients than shorter-acting ESAs. Confounding bias is a further bias inherent to this type of study. There are many confounding factors that can affect the Hb level over time, which do not necessarily reflect the efficacy of the treatment, such as the intensity of chemotherapy, iron supplementation or darbepoetin alfa dose adjustments or withholdings in response to Hb evolution. The national Romanian protocol for treatment of chemotherapy-induced anemia with ESAs recommends treatment of symptomatic anemia with a Hb in the range of 9–11 g/dL with a treatment Hb target of above 11 g/dL. Once the target Hb is reached, it is recommended to individualize treatment to maintain Hb with a minimum of treatment. However, the local prescription protocol in Bucharest changed in 2013, approximately one year after the study start, and allowed initiation of ESAs only in patients with a Hb in the range of 8–10 g/dL. Of the 35 participating centers, eight were in Bucharest, contributing a total of 118 patients (24% of the 497 patients in the FAS). Of the total study population, 263 patients (52.9%) were enrolled at a Hb of 8–10 g/dL and 77 patients (15.5%) at Hb < 9 g/dL. The Hb shift table shown in Table 4 shows that fewer patients with Hb < 9 g/dL at baseline achieved Hb > 10 g/dL or >11 g/dL, as was defined as the target Hb in the secondary endpoints. This finding suggests that initiating ESAs rather earlier than later within the recommended range of Hb 9–11 g/dL may improve target achievement. There are several other factors potentially confounding the results of Hb evolution and target achievement over time. Hb values may vary strongly by tumor type and type of chemotherapy administered, as well as by comorbidities and concomitant medications. The potential influence of tumor type and chemotherapy type has been addressed in a logistic regression model and data are provided in the online supplement. Concomitant medications and comorbidities have, however, not been addressed in a similar way in a logistic regression model.

In order to minimize risk of selection bias, the selection of study sites encompassed sites from every major region of Romania that are capable of satisfying the selection criteria. Every attempt was made to include sites that are representative of patients across the country and representative of the types of disease (both solid and hematological tumor types) prevalent in the region. Efforts were made to ensure that each site contributed an appropriate number of patients to the overall analysis by specifying minimum and maximum total enrollment per site. This observational study was monitored (full monitoring in a representative sample, monitoring of key data such as provision of informed consent, eligibility criteria, safety reports, etc. in all patients) to minimize information bias.

While the darbepoetin alfa label mentions several types of adverse reactions, such as immune system disorder (hypersensitivity), cardiac and vascular (hypertension, thromboembolic events including pulmonary embolism), skin and subcutaneous tissue disorders (rash/erythema), and other administration site disorders (injection site pain), we found a low rate of reported adverse events, despite the physicians’ obligation to report, either to Romanian National Drug Agency or to the study sponsor.

## Conclusions

In this single-arm, prospective, longitudinal, multicenter observational study, approximately 80% of patients with symptomatic chemotherapy-induced anemia had a Hb in the range of 9–11 g/dL prior to antianemic treatment with darbepoetin alfa. During treatment with darbepoetin alfa pre-existing anemia improved substantially, with patients experiencing a median increase in Hb of 0.9 g/dL between baseline and week 12; 57% of patients with Hb < 10 g/dL at baseline achieved Hb ≥ 10 g/dL and 46% of patients with Hb ≤ 11 g/dL at baseline achieved Hb > 11 g/dL at week 12. The results of this study do not alter the currently known benefit–risk balance of darbepoetin alfa.

## Caption Electronic Supplementary Material

Laboratory assessments at enrolment, an analysis of the influence of different factors on the primary and secondary outcomes, the proportion of patients with Hb level ≤ 10 g/dL at initiation of darbepoetin alfa, the time to achieve the Hb target level, as well as the proportion of patients receiving chemotherapy at full dose on schedule
