# Supplementary material for: APPLY: A prospective observational study of clinical practice patterns of darbepoetin alfa use in patients with chemotherapy-induced anemia in Romania
Source: Memo. 2018 Mar 2;11(2):144–51. doi: 10.1007/s12254-018-0390-4 (PMC6006232; doi:10.1007/s12254-018-0390-4)
Supplement: Supplementary file 1 — Laboratory assessments at enrolment, an analysis of the influence of different factors on the primary and secondary outcomes, the proportion of patients with Hb level ≤ 10 g/dL at initiation of darbepoetin alfa, the time to achieve the Hb target level, as well as the proportion of patients receiving chemotherapy at full dose on schedule [file 12254_2018_390_MOESM1_ESM.docx]

**Online Supplement**

**APPLY: A Prospective Aranesp® Observational Study in Patients with Chemotherapy-Induced Anemia**

Nicoleta Sorina Badelita; Nicoleta Mariana Berbec, Anda Natalia Ciuhu, Gabriel Doru Ghizdavescu, Alexandru Iordan, Cristina Neacsu, Dorel Ionel Popovici, Benonia-Elena Rosioru, Mariana Madalina Vasilica

**Table S1. Laboratory assessments at enrolment**

| Laboratory Test | N=497 |
| --- | --- |
| Ferritin Level (ng/ml) |  |
| N | 61 |
| Mean (SD) | 466.627 (573.3533) |
| Median (Q1, Q3) | 275.300 (128.000, 648.000) |
| Min – Max | 12.70 - 3263.00 |
| Missing | 436 |
| Folic Acid (ng/ml) |  |
| N | 2 |
| Mean (SD) | 9.050 (3.6204) |
| Median (Q1, Q3) | 9.050 (6.490, 11.610) |
| Min – Max | 6.49 - 11.61 |
| Missing | 495 |
| Total Bilirubin (mg/dl) |  |
| N | 267 |
| Mean (SD | 0.5649 (0.62708) |
| Median (Q1, Q3) | 0.4500 (0.3000, 0.6400) |
| Min – Max | 0.200 - 9.200 |
| Missing | 230 |
| Vitamin B12 (µg/dl) |  |
| N | 3 |
| Mean (SD) | 152.0 (95.27) |
| Median (Q1, Q3) | 206.0 (42.0, 208.0) |
| Min – Max | 42 - 208 |
| Missing | 494 |
| Reticulocyte Count (%) |  |
| N | 36 |
| Mean (SD) | 9.958 (11.5220) |
| Median (Q1, Q3) | 2.000 (1.100, 17.000) |
| Min – Max | 0.10 - 49.00 |
| Missing | 461 |

Max, maximum value; min, minimum value; n, number of patients with the given parameter; N - total number of patients in the group/subgroup; SD, standard deviation; Q1, lower quartile; Q3 upper quartile.

**Table S2. Analysis of influence of different factors on the primary and secondary outcomes**

| Outcome | Ratio | 95% CI | P-value |
| --- | --- | --- | --- |
| *Hb within the 9-11 g/dL range at initiation of darbepoetin alfa* |  |  |  |
| Tumor types:  Breast cancer vs other solid tumors | 2.145 | [0.607, 7.577] | 0.2358 |
| Gynecological tumors vs other solid tumors | 0.954 | [0.410, 2.219] | 0.9128 |
| Hodgkin's disease vs other solid tumors | 0.358 | [0.062, 2.073] | 0.2515 |
| Lung cancer vs other solid tumors | 0.917 | [0.454, 1.850] | 0.8081 |
| Multiple myeloma vs other solid tumors | 0.378 | [0.184, 0.777] | 0.0081 |
| Non-Hodgkin's lymphoma vs other solid tumors | 0.519 | [0.262, 1.025] | 0.0590 |
| Other hematological malignancies vs other solid tumors | 0.358 | [0.121, 1.053] | 0.0621 |
| *Hb concentration > 11.0 g/dL at week 6 of the study while concentration at enrollment <= 11.0 g/dL* |  |  |  |
| Gender male vs female | 1.721 | [1.070, 2.769] | 0.0252 |
| Tumor types:  Breast cancer vs other solid tumors | 6.194 | [2.644, 14.509] | <.0001 |
| Gynecological tumors vs other solid tumors | 1.775 | [0.855, 3.688] | 0.1239 |
| Hodgkin's disease vs other solid tumors | 3.188 | [0.504, 20.178] | 0.2181 |
| Lung cancer vs other solid tumors | 0.902 | [0.505, 1.611] | 0.7279 |
| Multiple myeloma vs other solid tumors | 1.337 | [0.677, 2.640] | 0.4035 |
| Non-Hodgkin's lymphoma vs other solid tumors | 2.279 | [1.239, 4.191] | 0.0080 |
| other hematological malignancies vs other solid tumors | 1.165 | [0.387, 3.512] | 0.7856 |
| *Hb concentration > 11.0 g/dL at week 9 of the study while concentration at enrollment <= 11.0 g/dL* |  |  |  |
| Type of chemotherapy:  5-fluoruracil/capecitabine vs platinum | 1.214 | [0.412, 3.579] | 0.7251 |
| Anthracyclines vs platinum | 3.050 | [1.588, 5.857] | 0.0008 |
| Irinotecan vs platinum | 0.445 | [0.050, 3.971] | 0.4686 |
| Other vs platinum | 1.692 | [0.930, 3.076] | 0.0849 |
| Oxaliplatin vs platinum | 1.484 | [0.600, 3.670] | 0.3929 |
| Monoclonal antibody-based regimens vs   platinum | 4.844 | [2.373, 9.890] | <0.0001 |
| Taxanes vs platinum | 2.127 | [1.173, 3.857] | 0.0130 |
| Taxanes and anthracyclines vs platinum | >999.999 | [<0.001, >999.999] | 0.9849 |
| *Hb concentration > 11.0 g/dL at week 12 of the study while concentration at enrollment <= 11.0 g/dL* |  |  |  |
| Type of chemotherapy:  5-fluoruracil/capecitabine vs platinum | 1.775 | [0.615, 5.125] | 0.2888 |
| Anthracyclines vs platinum | 4.057 | [2.088, 7.882] | <.0001 |
| Irinotecan vs platinum | 0.507 | [0.057, 4.537] | 0.5436 |
| Other vs platinum | 1.675 | [0.909, 3.085] | 0.0983 |
| Oxaliplatin vs platinum | 2.997 | [1.201, 7.477] | 0.0186 |
| Monoclonal antibody-based regimens vs  platinum | 3.573 | [1.775, 7.194] | 0.0004 |
| Taxanes vs platinum | 2.212 | [1.207, 4.053] | 0.0102 |
| Taxanes and anthracyclines vs platinum | >999.999 | [<0.001, >999.999] | 0.9848 |
| *Hb concentration >= 10.0 g/dL at week 6 of the study while concentration at enrollment < 10.0 g/dL* |  |  |  |
| None |  |  |  |
| *Hb concentration >= 10.0 g/dL at week 9 of the study while concentration at enrollment < 10.0 g/dL* |  |  |  |
| None |  |  |  |
| *Hb concentration >= 10.0 g/dL at week 12 of the study while concentration at enrollment < 10.0 g/dL* |  |  |  |
| None |  |  |  |
| *RBC or full blood Transfusion after 5 weeks of DA therapy* |  |  |  |
| None |  |  |  |
| *Chemotherapy at Full Dose on Schedule (FDOS)* |  |  |  |
| Tumor types:  Breast cancer vs other solid tumors | 0.524 | [0.236, 1.161] | 0.1114 |
| Gynecological tumors vs other solid tumors | 0.902 | [0.464, 1.751] | 0.7597 |
| Hodgkin's disease vs other solid tumors | 0.759 | [0.134, 4.290] | 0.7553 |
| Lung cancer vs other solid tumors | 0.860 | [0.489, 1.511] | 0.5991 |
| Multiple myeloma vs other solid tumors | 0.607 | [0.310, 1.192] | 0.1470 |
| Non-Hodgkin's lymphoma vs other solid tumors | 0.332 | [0.170, 0.651] | 0.0013 |
| Other hematological malignancies vs other solid tumors | 0.434 | [0.136, 1.388] | 0.1594 |

Note: Odds Ratio calculated from Logistic Regression. Stepwise regression methods were applied to select the variables in the logistic regression model (P<=0.1 to enter, P<=0.1 to stay). Variables eligible for inclusion in the model: age, gender, type of chemotherapy, tumor type. Ratios are not estimable whenever the confidence interval includes the text '>999.999'. Observations with missing model variables are excluded from this analysis. Only variables kept in the final model are presented in the table.

**Table S3. Proportion of patients with Hb level ≤ 10 g/dL at initiation of darbepoetin alfa**

| Covariates | n | N | % | 95% CI |
| --- | --- | --- | --- | --- |
| All patients | 269 | 497 | 54.1 | [49.6, 58.6] |
| Age |  |  |  |  |
| <65 | 174 | 319 | 54.5 | [48.9, 60.1] |
| >=65 | 95 | 178 | 53.4 | [45.8, 60.9] |
| Gender |  |  |  |  |
| Male | 142 | 255 | 55.7 | [49.4, 61.9] |
| Female | 127 | 242 | 52.5 | [46.0, 58.9] |
| Type of chemotherapy |  |  |  |  |
| Platinum | 61 | 109 | 56.0 | [46.1, 65.5] |
| Taxanes | 50 | 96 | 52.1 | [41.6, 62.4] |
| Anthracyclines | 36 | 70 | 51.4 | [39.2, 63.6] |
| Taxanes and anthracyclines | 0 | 1 | 0.0 | [ 0.0, 97.5] |
| Irinotecan | 2 | 6 | 33.3 | [ 4.3, 77.7] |
| Oxaliplatin | 11 | 27 | 40.7 | [22.4, 61.2] |
| 5-fluoruracil/capecitabine | 8 | 19 | 42.1 | [20.3, 66.5] |
| Monoclonal antibody-based regimens | 35 | 64 | 54.7 | [41.7, 67.2] |
| Other | 66 | 105 | 62.9 | [52.9, 72.1] |
| Tumor type |  |  |  |  |
| Lung cancer | 52 | 98 | 53.1 | [42.7, 63.2] |
| Breast cancer | 12 | 39 | 30.8 | [17.0, 47.6] |
| Gynecological tumors | 32 | 57 | 56.1 | [42.4, 69.3] |
| Other solid tumors | 67 | 145 | 46.2 | [37.9, 54.7] |
| Hodgkin's disease | 3 | 6 | 50.0 | [11.8, 88.2] |
| Multiple myeloma | 47 | 56 | 83.9 | [71.7, 92.4] |
| Non-Hodgkin's lymphoma | 45 | 78 | 57.7 | [46.0, 68.8] |
| Other hematological malignancies | 11 | 18 | 61.1 | [35.7, 82.7] |
| FDOS |  |  |  |  |
| Yes | 85 | 149 | 57.0 | [48.7, 65.1] |
| No | 169 | 318 | 53.1 | [47.5, 58.7] |

Note: percentages are based on the number of patients in full analysis set. Confidence intervals were obtained from the exact method.

FDOS, full dose on schedule

**Table S4. Time to achieve the Hb target level (days)**

| Target Hb Category | Statistics | N=497 |
| --- | --- | --- |
| >=10 g/dL[a] | n | 186 |
|  | Mean (SD) | 41.6 (39.57) |
|  | SE | 2.90 |
|  | Median (Q1, Q3) | 30.0 (21.0, 47.0) |
|  | Min | 1 – 298 |
| >11 g/dL[b] | n | 304 |
|  | Mean (SD) | 44.7 (34.99) |
|  | SE | 2.01 |
|  | Median (Q1, Q3) | 34.5 (21.0, 57.0) |
|  | Min - Max | 4 – 231 |

[a] only patients who had Hb concentrations at darbepoetin alfa initiation <10 g/dL are included in this section.

[b] only patients who had Hb concentrations at darbepoetin alfa initiation <=11 g/dL are included in this section.

Hb, hemoglobin; max, maximum value; min, minimum value; n, number of patients with the given parameter; N - total number of patients in the group/subgroup; SD, standard deviation; SE, standard error; Q1, lower quartile; Q3 upper quartile

**Table S5. Proportion of Patients receiving Chemotherapy at Full Dose on Schedule (FDOS)**

|  | n | N | % | 95% CI |
| --- | --- | --- | --- | --- |
| All Patients | 149 | 497 | 30.0 | [26.0, 34.2] |
| Age |  |  |  |  |
| <65 years | 98 | 319 | 30.7 | [25.7, 36.1] |
| >=65 years | 51 | 178 | 28.7 | [22.1, 35.9] |
| Gender |  |  |  |  |
| Male | 73 | 255 | 28.6 | [23.2, 34.6] |
| Female | 76 | 242 | 31.4 | [25.6, 37.7] |
| Type of chemotherapy |  |  |  |  |
| Platinum | 31 | 109 | 28.4 | [20.2, 37.9] |
| Taxanes | 31 | 96 | 32.3 | [23.1, 42.6] |
| Anthracyclines | 22 | 70 | 31.4 | [20.9, 43.6] |
| Taxanes and anthracyclines | 0 | 1 | 0.0 | [ 0.0, 97.5] |
| Irinotecan | 3 | 6 | 50.0 | [11.8, 88.2] |
| Oxaliplatin | 11 | 27 | 40.7 | [22.4, 61.2] |
| 5-fluoruracil/capecitabine | 6 | 19 | 31.6 | [12.6, 56.6] |
| Monoclonal antibody-based regimens | 11 | 64 | 17.2 | [8.9, 28.7] |
| Other | 34 | 105 | 32.4 | [23.6, 42.2] |
| Tumor type |  |  |  |  |
| Lung cancer | 30 | 98 | 30.6 | [21.7, 40.7] |
| Breast cancer | 10 | 39 | 25.6 | [13.0, 42.1] |
| Gynecological tumors | 19 | 57 | 33.3 | [21.4, 47.1] |
| Other solid tumors | 54 | 145 | 37.2 | [29.4, 45.7] |
| Hodgkin's disease | 2 | 6 | 33.3 | [4.3, 77.7] |
| Multiple myeloma | 16 | 56 | 28.6 | [17.3, 42.2] |
| Non-Hodgkin's lymphoma | 14 | 78 | 17.9 | [10.2, 28.3] |
| Other hematological malignancies | 4 | 18 | 22.2 | [6.4, 47.6] |

CI, confidence interval; n, number of patients with the given parameter; N - total number of patients in the group/subgroup
